# Layer specific observation of slow thermal equilibration in ultrathin metallic nanostructures by femtosecond X-ray diffraction

**DOI:** 10.1038/s41467-018-05693-5

**Published:** 2018-08-20

**Authors:** J. Pudell, A. A. Maznev, M. Herzog, M. Kronseder, C. H. Back, G. Malinowski, A. von Reppert, M. Bargheer

**Affiliations:** 1 Institut für Physik & Astronomie, Universität Potsdam, Karl-Liebknecht-Str. 24-25, 14476 Potsdam, Germany; 2 Department of Chemistry, Massachusetts Institute of Technology, Cambridge, MA 02139 USA; 3 Physics Department, Technical University Munich, 85748 Garching, Germany; 4 Institut für Experimentelle und Angewandte Physik, Universität Regensburg, 93040 Regensburg, Germany; 5 Institut Jean Lamour (UMR CNRS 7198), Université Lorraine, Vandœuvre-lès-Nancy, 54506 France; 6 Helmholtz-Zentrum Berlin for Materials and Energy GmbH, Wilhelm-Conrad-Röntgen Campus, BESSY II, Albert-Einstein-Str. 15, 12489 Berlin, Germany

## Abstract

Ultrafast heat transport in nanoscale metal multilayers is of great interest in the context of optically induced demagnetization, remagnetization and switching. If the penetration depth of light exceeds the bilayer thickness, layer-specific information is unavailable from optical probes. Femtosecond diffraction experiments provide unique experimental access to heat transport over single digit nanometer distances. Here, we investigate the structural response and the energy flow in the ultrathin double-layer system: gold on ferromagnetic nickel. Even though the excitation pulse is incident from the Au side, we observe a very rapid heating of the Ni lattice, whereas the Au lattice initially remains cold. The subsequent heat transfer from Ni to the Au lattice is found to be two orders of magnitude slower than predicted by the conventional heat equation and much slower than electron–phonon coupling times in Au. We present a simplified model calculation highlighting the relevant thermophysical quantities.

## Abstract (web summary)

Heat transport in ultrathin metal layers is important for potential applications in optical‐magnetic switching, but difficult to access experimentally. Here, the authors use ultrafast X‐ray diffraction to directly probe and explain unexpected time‐dependent transport behavior in Au–Ni nanolayers.

## Introduction

Ultrafast heating and cooling of thin metal films has been studied extensively to elucidate the fundamentals of electron–phonon interactions^1–7^ and heat transport at the nanoscale^8–13^. The energy flow in metal multilayers following optical excitation attracted particular attention in the context of heat-assisted magnetic recording^14,15^ and all-optical magnetic switching^16–18^. The role of temperature in optically induced femtosecond demagnetization is intensely discussed, particularly with regard to multipulse switching scenarios^19^. Two- or three-temperature models (TTMs) are often used to fit the experimental observations^20^. The microscopic three-temperature model (M3TM)^20^, which uses Elliot–Yafet spin-flip scattering as the main mechanism for ultrafast demagnetization is often contrasted against superdiffusive spin transport^21^. Such electron transport is closely related to ultrafast spin-Seebeck effects^22,23^, which require a description with independent majority and minority spin temperatures. The heat flow involving electrons, phonons, and spins has been found to play a profound role in ultrafast magnetization dynamics^24,25^. The description of the observed dynamics in TTMs or the M3TM are challenged by ab initio theory which explicitly holds the nonequilibrium distribution responsible for the very fast photoinduced demagnetization^26,27^. The presence of multiple subsystems (lattice, electrons, and spins), e.g., in ferromagnetic metals^5,28^, poses a formidable challenge for experimental studies of their coupling and thermal transport on ultrafast time scales when these subsystems are generally not in equilibrium with each other^26,27,29^. Temperature dynamics in metal films are typically monitored using optical probe pulses via time-domain thermoreflectance (TDTR)^12^. This technique has been a workhorse of nanoscale thermal transport studies, but experiences significant limitations when applied to ultrathin multilayers with individual layer thicknesses falling below the optical skin depth, which are in the focus of ultrafast magnetism research^22–24,30–32^. Optical probes are generally sensitive to electronic and lattice temperatures, although in some cases the lattice temperature^13^ or the spin temperature^24^ may be deduced. In order to understand the thermal energy flow, it is highly desirable to directly access the temperature of the lattice, which provides the largest contribution to the specific heat. Ultrafast X-ray diffraction is selectively sensitive to the crystal lattice, and material-specific Bragg angles enable measurements of multiple layers even when they are thinner than the optical skin depth and/or buried below opaque capping layers^33–35^. The lattice constant variations of each layer can be measured with high absolute accuracy, making it possible to determine the amount of deposited heat in metal bilayers that was debated recently^30–32^. The great promise of ultrafast X-ray diffraction (UXRD) for nanoscale thermal transport measurements and ultrafast lattice dynamics has already been demonstrated in experiments with synchrotron-based sources^33–36^. However, limited temporal resolution of these experiments (~100 ps) only allowed to study heat transport on a relatively slow (nanosecond) time scale and over distances >100 nm. Ultrafast nanoscale thermal transport research will greatly benefit from femtosecond X-ray sources. While free electron laser facilities are in very high demand, an alternative is offered by laser-based plasma sources of femtosecond X-rays^37,38^, which lack the coherence and high flux of a free electron laser but are fully adequate for UXRD measurements^6,39,40^. As an example, a recent experiment on 6 nm thick Au nanotriangles^39^ confirmed the $\tau _{{\mathrm{Au}}}^0$ = 5 ps electron–phonon equilibration time generally accepted for high fluence excitation of Au^3,6,41,42^. For similar fluences ultrafast electron diffraction reported $\tau _{{\mathrm{Ni}}}^0$ = 0.75 to 1 ps for Ni thin films between room temperature and Curie temperature *T*_C_^7,43^.

**Fig. 1:**
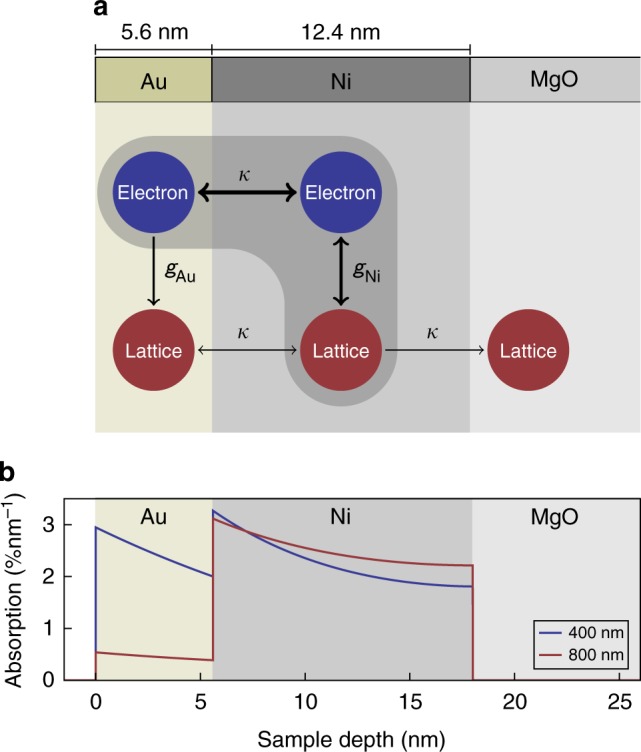
Schematic of heat reservoirs in the sample structure. **a** Layer stacking of the metallic heterostructure: Au on Ni deposited on an MgO substrate. Each layer has a phonon heat reservoir. The metal layers additionally have an electronic heat reservoir. The heat contained in the Ni spin system is included in the electron system. The electron–phonon coupling constants *g*_Au_ and *g*_Ni_ parametrize the local energy flow among electrons and phonons within each layer, whereas the thermal conductivity *κ* indicates spatial heat transport. **b** Calculated optical absorption profiles in the metallic bilayer

In this report, we demonstrate that the use of a femtosecond X-ray probe enables thermal transport measurements over a distance as small as ~5 nm in a Au/Ni bilayer with thickness *d*_Au_ = 5.6 nm and *d*_Ni_ = 12.4 nm grown on MgO. By monitoring the dynamics of the lattice constants of Au and Ni, we find that the Ni lattice fully expands within about 2 ps, while the Au lattice initially remains cold even if a significant fraction of the excitation light is absorbed by the electronic subsystem in Au. The Au lattice then heats up slowly, reaching the maximum temperature about 80 ps after the optical excitation. The observed thermal relaxation of the bilayer structure is two orders of magnitude slower than 1 ps predicted by the heat equation and also much slower than the usual electron–phonon equilibration time $\tau _{{\mathrm{Au}}}^0$ = 1–5 ps (see Table 1)^3,41,42^. We explain this surprising result in a model (see Fig. 1) based on the keen insight into the physics of the thermal transport in Au–Pt bilayers offered in recent studies^11,13^, which showed that nonequilibrium between electrons and lattice in Au persists for a much longer time in a bilayer than in a single Au film. We find, furthermore, that on the spatial scale of our experiment thermal transport by phonons in metals can no longer be neglected. Our results underscore challenges for thermal transport modeling on the nanometer scale. On the other hand, they demonstrate the great potential of the UXRD for monitoring thermal transport under experimental conditions typical for studies of ultrafast magnetism^20,44^.

**Table 1:** Thermophysical parameters of Au and Ni

Parameter	Gold	Nickel
Lattice specific heat, *C*^ph^ (10^6^ Jm^−3^ K^−1^)	2.5^54^	3.8^55^
Sommerfeld constant, *γ*^S^ (Jm^−3^ K^−2^)	67.5^2^	1074^2^
Electron–phonon coupling constant, *g* (10^16^ Wm^−3^ K^−1^)	1–4^2^	36–105^2^
e–ph coupling time isolated layers @1000 K, *τ*^0^ (ps)	1.7–6.7	1–3
e–ph coupling time equilibrated electrons @1000 K, *τ* (ps)	26–107	1–3
Thermal conductivity, *κ* (Wm^−1^ K^−1^)	318^56^	90^56^
Thermal conductivity (lattice), *κ*^ph^ (Wm^−1^ K^−1^)	5^56^	9.6^56^
Expansion coefficient with Poisson correction, *α*^uf^ (10^−5^ K^−1^)	3.16^57^	2.8^57^

Literature values for material parameters relevant for modeling the heat transfer after laser excitation. For *C*^ph^ we use the parameters at room temperature. The e–ph coupling time ranges are calculated for 1000 K to show that for an equilibrated electron system, the e–ph coupling time in Ni is much shorter than in Au

## Results

### Experiment

We use femtosecond laser pulses at 400 and 800 nm to excite the electron system of Au and Ni through the Au top layer. The sample structure and the calculated absorption profiles are shown in Fig. 1. We note that for 400 nm pulses the absorbed energy density $\rho _{{\mathrm{Au,Ni}}}^Q$ in Au and Ni is similar, whereas for 800 nm almost no light is absorbed in Au. The much higher absorption of 400 nm light in Au is a result of the larger real part of the refractive index^31,32^. For our 5.6 nm thick Au film, the destructive interference of light reflected at the interfaces additionally contributes to the suppressed absorption at 800 nm.

**Fig. 2:**
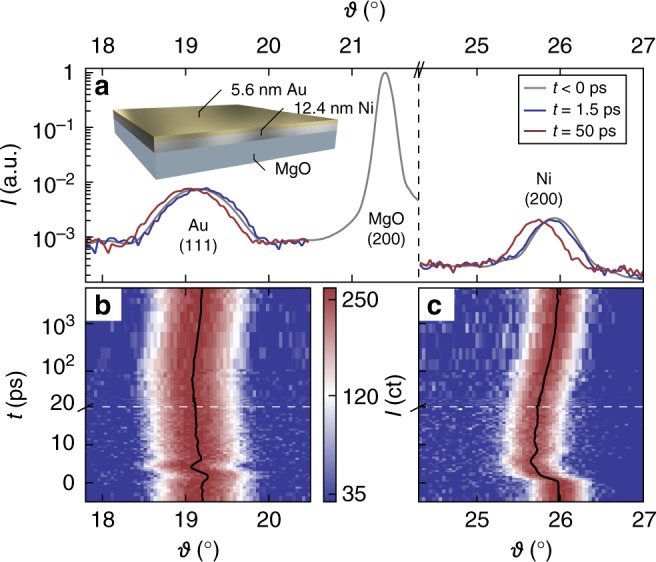
Experimental data. **a** X-ray diffraction pattern of the sample (see inset) evidencing the crystalline orientation of the Au and Ni nanolayers. Colored lines visualize transient shifts of the Bragg peaks at selected times. Their full time evolution is shown in panels (**b**) for Au and **c** for Ni along with the respective peak center positions (black line). The white dashed line indicates the axis break from linear to logarithmic time scale

The strains *ε*_Au,Ni_ determined via Bragg’s law from UXRD data (Fig. 2b, c) can be converted to lattice temperature changes Δ*T*_Au,Ni_ and energy density changes $\rho _{{\mathrm{Au,Ni}}}^Q$ via

1 $$\varepsilon _{{\mathrm{Au,Ni}}} = \alpha _{{\mathrm{Au,Ni}}}^{{\mathrm{uf}}}{\mathrm{\Delta }}T_{{\mathrm{Au,Ni}}}$$

2 $$\varepsilon _{{\mathrm{Au,Ni}}} = \frac{{\alpha _{{\mathrm{Au,Ni}}}^{{\mathrm{uf}}}}}{{C_{{\mathrm{Au,Ni}}}}}\rho _{{\mathrm{Au,Ni}}}^Q$$

using effective out-of-plane expansion coefficients $\alpha _{{\mathrm{Au,Ni}}}^{{\mathrm{uf}}}$ and specific heats *C*_Au,Ni_, which are generally temperature dependent. For our experimental conditions temperature-independent coefficients are good approximations. The effective expansion coefficients $\alpha _{{\mathrm{Au,Ni}}}^{{\mathrm{uf}}}$ take into account the crystalline orientation of the films and the fact that on ultrafast (uf) timescales the film can exclusively expand out-of plane, since the uniform heating of a large pump-spot region leads to a one-dimensional situation, as in-plane forces on the atoms by the thermal stresses vanish. For details about $\alpha _{{\mathrm{Au,Ni}}}^{{\mathrm{uf}}}$ and a description how heat in electrons and phonons drive the transient stress via macroscopic Grüneisen coefficients see the Methods section.

**Fig. 3:**
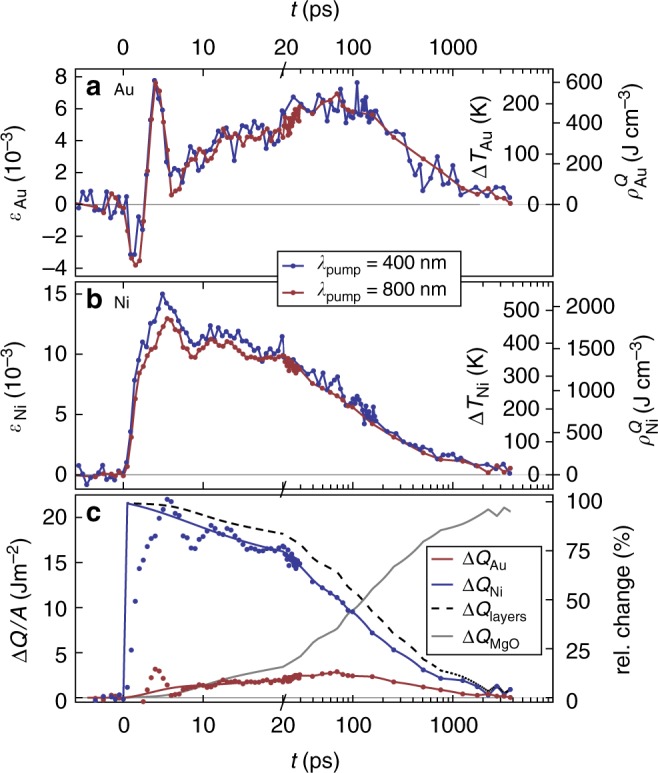
Transient energy densities and temperatures. Transient lattice strain *ε* in the Au film (**a**) and the Ni film (**b**) as measured by UXRD after excitation with 400 nm (blue) and 800 nm (red) light pulses. The right axis label the temperature change Δ*T* and the energy density *ρ*^*Q*^ calculated from *ε*. **c** Red and blue dots show the energy per unit area Δ*Q*/*A* obtained from (**a**, **b**) by multiplication with *d*_Au,Ni_. The red and blue lines show thermal dynamics with acoustic oscillations removed, yielding the true energy per unit area Δ*Q*/*A*. The black dashed line shows the sum of these energies. The gray line is the thermal energy that has been transported into the substrate

We now discuss the information that can be directly inferred from the measured transient strains (Fig. 3) in the laser-excited metallic bilayer without any advanced modeling. For convenience, we added two right vertical axes to Fig. 3a, b showing the layer-specific temperature and energy density according to Eqs. (1) and (2). Initially Ni expands, while the Au layer gets compressed by the expansion of the Ni film. Around 3 ps Au shows a pronounced expansion, when the compression wave turns into an expansion wave upon reflection at the surface. Less pronounced signatures of the strain wave are observed in Ni as well. A surprisingly long time of about 80 ps is required to reach the maximum expansion of Au by transport of heat from the adjacent Ni until *T*_Au_ ≈ *T*_Ni_. For times *t* > 100 ps, cooling by heat transfer to the substrate dominates the signal. In Fig. 3c we show the heat energy Δ*Q*_MgO_ flowing through a unit area *A* into the substrate, which we can directly calculate from the measured energy densities via

3 $${\mathrm{\Delta }}Q_{{\mathrm{MgO}}}(t){\mathrm{/}}A = - d_{{\mathrm{Au}}}{\mathrm{\Delta }}\rho _{{\mathrm{Au}}}^Q(t) - d_{{\mathrm{Ni}}}{\mathrm{\Delta }}\rho _{{\mathrm{Ni}}}^Q(t).$$

${\mathrm{\Delta }}\rho _{{\mathrm{Ni,Au}}}^Q(t)$ = $\rho _{{\mathrm{Ni,Au}}}^Q(t) - \rho _{{\mathrm{Ni,Au}}}^Q(0)$ are the changes of the energy densities $\rho _{{\mathrm{Ni}}}^{{Q}}$ and $\rho _{{\mathrm{Au}}}^{{Q}}$ with respect to the initially deposited energy densities. Even when the temperatures are equilibrated at *t* > 100 ps, $\rho _{{\mathrm{Ni}}}^{{Q}}$ and $\rho _{{\mathrm{Au}}}^{{Q}}$ differ strongly because of the different specific heat of Au and Ni. Figure 3c confirms that within the first 20 ps the heat energy Δ*Q*_Au_ = $d_{{\mathrm{Au}}}{\mathrm{\Delta }}\rho _{{\mathrm{Au}}}^{{Q}}$ flowing from Ni into Au is similar to the amount Δ*Q*_MgO_ transported into the substrate. At about 150 ps half of the energy deposited in the film has been transported into the substrate. However, leaking a fraction of the thermal energy to the insulating substrate does not explain why the ultrathin Au layer is not much more rapidly heated via electronic heat transport typical of metals.

### Modeling

Inspired by the recent studies using TDTR^11,13^ we set up a modified two-temperature model graphically represented in Fig. 1b to rationalize the slow Au heating observed in Fig. 3a. We first justify this simplified modeling. The high electron conductivity—potentially including ballistic and superdiffusive electrons—rapidly equilibrates the electron systems of Ni and Au. The fact that the Au layer is equally compressed in the first 2 ps irrespective of the excitation wavelengths is an experimental proof of the rapid equilibration of electron temperatures. Otherwise the high electron pressure in Au after 400 nm excitation (cf. Fig. 1c) would counterbalance the compression caused by the Ni expansion^6^. As Ni has a much larger Sommerfeld constant (Table 1) the electronic specific heat *C*^e^ = *γ*^S^*T* is dominated by Ni and the ratio of energy densities $\rho _{{\mathrm{Ni}}}^{{Q}}{\mathrm{/}}\rho _{{\mathrm{Au}}}^{{Q}}$ ≈ 10 is large at 1 ps. A significant electronic interface resistance^45^ that would prevent a rapid equilibration of electron temperatures in Au and Ni is clearly incompatible with our measurements at 400 nm. If the electrons did not equilibrate much faster than 1 ps and effectively remove the heat deposited in the electron system of Au, we would not observe the same strong compression of the Au lattice, since electronic pressure would instantaneously force the Au to expand^6,7,40,43^. In the diffuse-mismatch model, the electronic interface conductance of metals increases linearly with the temperature and can be calculated from the Sommerfeld constant and the Fermi velocity^45^. Immediately after excitation, the electron temperature reaches several thousand Kelvin, which leads to a subpicosecond thermalization of the electrons in simulations, including the interface resistance.

The electron–phonon coupling constant in Ni is much larger than in Au (Table 1). Consequently, nearly all photon energy initially absorbed in the electronic system is funneled into the Ni lattice, even when one third of the absorbed energy is initially deposited in the electronic system of Au with 400 nm excitation. In contrast, the electron–phonon coupling times $\tau _{{\mathrm{Au,Ni}}}^0$ = $C_{{\mathrm{Au,Ni}}}^{\mathrm{e}}{\mathrm{/}}g_{{\mathrm{Au,Ni}}}$ for Au and Ni are not very different if the films are not in contact, because the large electronic specific heat $C_{{\mathrm{Ni}}}^{\mathrm{e}}$ of Ni cancels its large electron–phonon coupling constant *g*_Ni_ (see Table 1). However, in the bilayer, the electrons in Au and Ni rapidly form an equilibrated heat bath with $C_{{\mathrm{tot}}}^{\mathrm{e}} \approx C_{{\mathrm{Ni}}}^{\mathrm{e}}$. Now only the electron–phonon coupling constant determines the coupling time: *τ*_Ni_ = $C_{{\mathrm{tot}}}^{\mathrm{e}}{\mathrm{/}}g_{{\mathrm{Ni}}} \ll C_{{\mathrm{tot}}}^{\mathrm{e}}{\mathrm{/}}g_{{\mathrm{Au}}}$ = *τ*_Au_.

We start the numerical modeling when a quasi-equilibrium temperature in the combined system *C*_com_ = $C_{\mathrm{Au}}^{\mathrm{e}} + C_{\mathrm{Ni}}^{\mathrm{e}} + C_{\mathrm{Ni}}^{\mathrm{ph}} \approx$ $C_{\mathrm{Ni}}^{\mathrm{e}} + C_{\mathrm{Ni}}^{\mathrm{ph}}$ ≈ *C*_Ni_ is established after electron–phonon equilibration in Ni around *τ*_Ni_ = $C_{{\mathrm{tot}}}^{\mathrm{e}}{\mathrm{/}}g_{{\mathrm{Ni}}} \approx C_{{\mathrm{Ni}}}^{\mathrm{e}}{\mathrm{/}}g_{{\mathrm{Ni}}}$ ≈ 1 ps. Since $C_{{\mathrm{Ni}}}^{{\mathrm{ph}}} \gg C_{{\mathrm{Ni}}}^{\mathrm{e}} \gg C_{{\mathrm{Au}}}^{\mathrm{e}}$ and *d*_Ni_ > *d*_Au_, we refer to the combined system as *C*_Ni_ in the equations. Since the energy stored in each layer is proportional to their thickness and the energy transfer rate from electrons to phonons in Au is proportional to the Au volume *V*_Au_ ∝ *d*_Au_, the differential equations describing this special TTM represented in Fig. 1b read

4 $$d_{{\mathrm{Au}}}C_{{\mathrm{Au}}}^{{\mathrm{ph}}}\frac{{\partial T_{{\mathrm{Au}}}^{{\mathrm{ph}}}}}{{\partial t}} = d_{{\mathrm{Au}}}g_{{\mathrm{Au}}}\left( {T_{{\mathrm{Ni}}} - T_{{\mathrm{Au}}}^{{\mathrm{ph}}}} \right)$$

5 $$d_{{\mathrm{Ni}}}C_{{\mathrm{Ni}}}\frac{{\partial T_{{\mathrm{Ni}}}}}{{\partial t}} = d_{{\mathrm{Au}}}g_{{\mathrm{Au}}}\left( {T_{{\mathrm{Au}}}^{{\mathrm{ph}}} - T_{{\mathrm{Ni}}}} \right).$$

Note that the two temperatures in this model are the temperature of the Au lattice, $T_{{\mathrm{Au}}}^{{\mathrm{ph}}}$ and the temperature of the combined system, which is denoted as *T*_Ni_, keeping in mind that this Ni temperature equals the Au electron temperature. For small temperature changes over which the specific heats are approximately constant, the solution to this system of equations is an exponential decay of *T*_Ni_ ~ *e*^−*t*/*τ*^ and a concomitant rise of the Au lattice temperature *T*_Au_ ~ (1 − *e*^−*t*/*τ*^) on the characteristic timescale

6 $$\tau = \frac{1}{{g_{{\mathrm{Au}}}\left( {\frac{1}{{C_{{\mathrm{Au}}}}} + \frac{{d_{{\mathrm{Au}}}}}{{d_{{\mathrm{Ni}}}}}\frac{1}{{C_{{\mathrm{Ni}}}}}} \right)}}.$$

Due to the small film thickness and the rapid electronic heat diffusion, we do not assume any gradient in the temperatures of each film. At about 1 ps after excitation we define the initial conditions as $T_{{\mathrm{Ni}}}(1{\kern 1pt} {\mathrm{ps}}) = T_{{\mathrm{Ni}}}^{\mathrm{i}}$ and $T_{{\mathrm{Au}}}^{\mathrm{i}} \approx 0$. The final temperature after equilibrating the temperatures of the two thin films, neglecting heat transport to the substrate is

7 $$T^{\mathrm{f}} = T^{\mathrm{i}}\frac{{d_{{\mathrm{Ni}}}C_{{\mathrm{Ni}}}}}{{d_{{\mathrm{Au}}}C_{{\mathrm{Au}}}^{{\mathrm{ph}}} + d_{{\mathrm{Ni}}}C_{{\mathrm{Ni}}}}}.$$

**Fig. 4:**
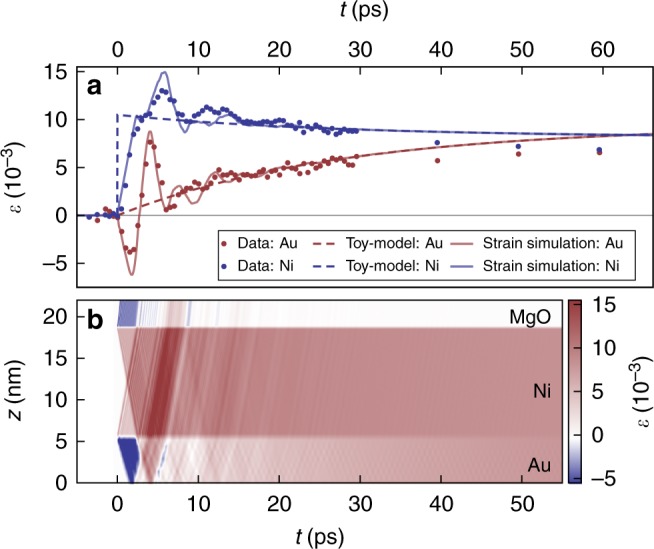
Comparison of models with the experimental data. **a** Dots indicate the measured strain *ε*. The dashed lines represent the strain calculated from the average heating of the layers according to the model visualized in Fig. 1b. Solid lines are simulations, which are based on this model and additionally include the strain waves triggered by the impulsive excitation (see Methods section). Heat transport to the substrate is not included. **b** Color-coded strain *ε* as a function of sample depth and time *t*, which is simulated assuming a spatially homogeneous transient thermal stress in each layer which is proportional to the dashed lines in (**a**). Spatial averaging of the strain *ε*(*t*) in each layer yields the solid lines in panel (**a**)

This very simple model (dashed lines of Fig. 4a) for the transient quasi-equilibrium temperatures agrees very well with the data. In particular, the exponential rise of *T*_Au_ and the exponential decay of *T*_Ni_ converge around 80 ps. Deviations at longer times originate mainly from heat transport into the MgO substrate, which is not included in the model (dashed lines).

The only fitting parameters of our model are the initial temperature *T*^i^ and the electron–phonon coupling constant of Au. With our simple model we get the best fit using *g*_Au_ = 6.5 × 10^16^ W m^−3^ K^−1^, which is somewhat larger than the range from 1 to 4 × 10^16^ W m^−3^ K^−1^ reported in the literature^2,3^. If—as an example—we reduce the electron–phonon coupling constant to the value of 4 × 10^16^ W m^−3^ K^−1^, the calculated equilibration of *T*_Au_ and *T*_Ni_ is much too slow. Including electronic interface resistance would make it even slower. The missing energy transfer rate, however, can be easily rationalized by phonon heat conductivity *κ*^ph^. If we fully disregarded electronic heat conduction in Au, the literature value for $\kappa _{{\mathrm{Au}}}^{{\mathrm{ph}}}$ given in Table 1 would lead to an equilibration of Au and Ni temperature exclusively via phonons three times faster than we observe. The phonon heat transport is probably much less efficient than this prediction because of additional interface resistances for phonon heat transport and because the mean free path of phonons is on the order of the layer thickness^8,10^. However, we do not attempt to quantify *κ*^ph^ and *g*_Au_ here. We only note qualitatively that to conform to the expected values of electron–phonon coupling in Au, the phonon heat conduction must become important in nanoscale multilayers, even though normally the heat conduction in metals is dominated by electrons ($\kappa \gg \kappa ^{{\mathrm{ph}}}$ see Table 1). Phonon heat transport is not included in our numerical calculations, because in fact the heat diffusion equation is not valid at such small length scales below the phonon mean free path. Similarly, a complex theoretical modeling would be required to simulate the heat transport to the substrate, e.g., by heat transfer from Ni electrons to MgO phonons at the interface^46^. Figure 3c provides a benchmark of the experimentally determined phonon heat transport into the substrate.

## Discussion

In summary, the modified TTM model (Eqs. (4) and (5)) captures the essence of heat transport between ultrathin metal films: the electrons in Au and Ni are rapidly equilibrated. This is evidenced by the fact that 400 and 800 nm excitation both initially only heat Ni, regardless of the energy absorbed in Au. For 400 nm excitation we showed an intricate process of shuttling heat energy back and forth between the layers: the electrons first rapidly transport energy from Au into Ni (e–e equilibration $\ll 1$ ps) before they transport some of the heat back from the Ni phonons to the Au phonons. Finally, the heat flows back through Ni toward the substrate. Heat transport by phonons can account for a fraction of the Au heating. The energy transported from the Ni phonons via Ni and Au electrons into the Au lattice is throttled by the weak electron–phonon coupling in Au. We believe that our results will have an important impact on ultrafast studies of the spin-Seebeck effect, superdiffusive electron transport as well as optical demagnetization and remagnetization. Precise measurements of the total heat in the system after few picoseconds will help to determine the actually required laser fluence in ultrafast demagnetization studies, which currently diverge by an order of magnitude in the literature^44,47^. The lattice is not only discussed as the sink of angular momentum in the ultrafast demagnetization: with its dominant heat capacity the lattice constitutes the heat bath which controls the speed of reordering of the spin systems at high fluence^20,44^. Our detailed account of heat flow in Ni after photo-excitation must influence the interpretation of MOKE data, which were fitted in previous studies^20,48^ by using a value for the specific heat of the Ni phonon system which is a factor of two below the Dulong–Petit value.

We have demonstrated the power of UXRD in probing nanoscale heat transport in an ultrathin metallic bilayer system which is relevant to current magnetic recording developments such as heat-assisted magnetic recording. To understand the all-optical-^15^ and helicity-dependent^49^ switching in ferrimagnets and two different timescales observed in the demagnetization of transition metals^20,44^ or rare earths^50,51^, precise calibration of the lattice temperature is crucial. We are convinced that the direct access to the lattice, the layer-specific information for layers thinner than the optical skin depth, the conceptual simplicity of the arguments and the experimental geometry make the paper particularly useful for comparisons to previous^20,30–32,44^ and future work on optical manipulation of spins.

## Methods

### Sample growth and UXRD

Ni/Au stacks with different Ni and Au thicknesses were grown by molecular beam epitaxy onto a MgO(001) substrate at 100 °C. The MgO(001) substrates were degassed at 350 °C for 10 min. The pressure during growth never exceeded 6^−10^ mbar. We measured the layer thicknesses *d*_Au_ = 5.6 nm and *d*_Ni_ = 12.4 nm of the investigated sample by X-ray reflectivity. The 24 lattice planes of Au yield a symmetric (111) Bragg reflection (Fig. 2a) at *ϑ* = 19.29°, well separated from the symmetric (200) Ni peak at 25.92° originating from 70 lattice planes. The lattice strains *ε*_Ni,Au_(*t*) = −cot(*ϑ*(*t*))Δ*ϑ*(*t*) perpendicular to the sample surface are directly retrieved from the time-resolved Bragg-peak positions *ϑ*(*t*) (Fig. 2b, c)^58,39,40^. These UXRD data were recorded at our laser-driven plasma X-ray source at the University of Potsdam, that emits 200 fs X-ray pulses with a photon energy of 8 keV. The sample was excited by p-polarized 400 and 800 nm laser pulses of about 100 fs duration with a pulse energy of 0.3 mJ and a diameter of 1.5 mm (FWHM). Since the angle between the pump pulse and the Bragg-reflecting X-ray probe pulse is fixed in the setup, we take into accout the modified angle of incidence of the optical pulse of 44° (51°) with respect to the surface normal for the Ni (Au) reflection to calculate the incident fluence of 9 (8) mJ/cm^2^ and an absorbed fluence of 3 (2.9) mJ/cm^2^ for our bilayer system using a matrix formalism, which also yields the absorption profiles at 400 and 800 nm excitation shown in Fig. 1c^52^. The above values are for 800 nm excitation, and the 400 nm data in Fig. 3 are scaled up for better comparison of the two different excitation conditions.

### Correction of the thermal expansion coefficient

The effective expansion coefficient $\alpha _{{\mathrm{Au,Ni}}}^{{\mathrm{uf}}}$ valid for heating a thin epitaxial layer is based on the lattice constants and strains predicted from equilibrium thermal expansion coefficients, corrected according to the Poisson effect^53^. In cubic materials with (100) surface orientation the ratio of the observed ultrafast (uf) strain and the strain *ε*^eq^ = *α*^eq^(*T*)Δ*T* along the (100) direction calculated from equilibrium value (eq) is *ε*/*ε*^eq^ = *α*^uf^(*T*)/*α*^eq^(*T*) = 1 + 2*C*_12_/*C*_11_ = 2.2 for Ni and would be 2.6 for Au. For the Au (111) cubic crystal surface, the above equation is still valid if the elastic constants are calculated in the rotated coordinate system, in which the *x*-axis is [111]. We find that the newly obtained *C*_11_ and *C*_12_ coincidentally yield the same correction factor of 2.2 for Au (111) as for Ni (100).

### Strain waves prove ultrafast electron-equilibration

The pronounced compression and expansion of the Au layer (see Fig. 4a) clearly originates from the laser-induced stress generated in Ni. In order to show that our modified TTM predicting negligible energy density in Au immediately after the excitation can quantitatively explain the signal oscillations, we have used the transient temperatures *T*_Ni,Au_(*t*) from our TTM as input parameters for a full thermo-elastic simulation using the udkm1Dsim toolbox, which are represented as solid lines in Fig. 4a^59^. For convenience, Fig. 4b shows the spatio-temporal strain map from which the solid lines in Fig. 4a are calculated by spatial averaging over the layer for each time delay. Multiple reflections of strain waves at the interfaces are strongly damped by transmission to the substrate.

### Macroscopic Grüneisen coefficients

Several recent ultrafast X-ray diffraction and electron diffraction experiments on thin metal films have highlighted two contributions of electrons and phonons to the transient stress *σ*, which drives the observed strain waves. A very useful concept uses the macroscopic Grüneisen coefficient Γ^e^ and Γ^ph^, which relate the energy densities *ρ*^*Q*^ to the stress *σ* = Γ*ρ*. While in Au the electronic Grüneisen constant ${\mathrm{\Gamma }}_{{\mathrm{Au}}}^{\mathrm{e}}$ = 1.5 is about half of its phonon counterpart $\Gamma _{{\mathrm{Au}}}^{{\mathrm{ph}}}$ = 3.0, in Ni ${\mathrm{\Gamma }}_{{\mathrm{Ni}}}^{\mathrm{e}}$ = 1.5 is only slightly different from ${\mathrm{\Gamma }}_{{\mathrm{Ni}}}^{{\mathrm{ph}}}$ = 1.7^6,7^. For our analysis the distinction of the origin of pressure in Ni is not very relevant, since the redistribution of energy from electrons to phonons only increases the stress by 15%. In Au the electron pressure is negligible in our bilayer system, since due to the large electronic specific heat of Ni and the subpicosecond equilibration among the electrons, all the energy is accumulated in Ni. The ab initio modeling discussed in connection to the recent UXRD study on Fe points out that both electron–phonon coupling parameters and phonon Grüneisen coefficients depend on the phonon mode^5,29^. While in that study the scattering of X-rays from individual phonon modes selected by the scattering geometry may require a mode-specific analysis, we believe that measuring the lattice expansion via a Bragg-peak shift looks at an average response of the lattice to all phonon modes, and hence a mode-averaged analysis is reasonable if there is no selective excitation of modes with extraordinarily different Grüneisen coefficients.

### Data availability

The data that support the findings of this study are available from the corresponding authors on reasonable request.
